# Integrating mental health and psychosocial support (MHPSS) into infectious disease outbreak response: Results of an expert consensus study

**DOI:** 10.1016/j.ijregi.2024.100396

**Published:** 2024-06-26

**Authors:** Biksegn Asrat Yirdaw, Marianne Moussallem, Muhammad Alkasaby, Michele Kosremelli Asmar, Vania Alves, Dumsani Njobo Mamba, Basma Tolba, Claire W. Kyalo, Mujataba Hussain, Jennifer Dafwat, Godfrey Kagaayi, Duncan Nkhoma, Julian Eaton, Ian F. Walker

**Affiliations:** 1 UK Public Health Rapid Support Team, UK Health Security Agency/London School of Hygiene & Tropical Medicine, London, UK; 2 Centre for Global Mental Health, London School of Hygiene & Tropical Medicine, London, UK; 3 Higher Institute of Public Health, Saint-Joseph University of Beirut, Beirut, Lebanon; 4 Child Protection in Humanitarian Action, UNICEF, New York, USA; 5 Africa Centres for Disease Control and Prevention, Addis Ababa, Ethiopia; 6 High Institute of Public Health, Alexandria University, Alexandria, Egypt; 7 Global Mental Health Peer Network, Nairobi, Kenya; 8 Child Psychiatry Unit, Institute of Mental Health and Neurosciences, Kashmir, India; 9 Global Mental Health Peer Network, Country Lead, Abuja, Nigeria; 10 Movement for Global Mental Health, Kampala, Uganda; 11 Global Mental Health Peer Network, Lusaka, Zambia; 12 CBM Global, Cambridge, UK; 13 Office for Health Improvement and Disparities, Department of Health and Social Care, London, UK

**Keywords:** Mental health and psychosocial support, Infectious disease outbreaks, Public health, Integrated outbreak response, Lived experience

## Abstract

**Objectives:**

Outbreaks are associated with increased risk of anxiety disorders, depression, and severe mental conditions. Integrating mental health and psychosocial support (MHPSS) into outbreak response facilitates the delivery of holistic care to the affected community. As there is an increasing incidence of outbreaks globally, integrating MHPSS into preparedness and response plans is paramount to strengthen the capacity of existing health systems and respond to mental health and psychosocial needs. However, the attention given to MHPSS during outbreak response is critically low. The objectives of this study were to identify areas of MHPSS integration and explore the challenges that hinder the delivery of an integrated care during outbreak response.

**Methods:**

A participatory qualitative study was conducted to explore how MHPSS can be incorporated into outbreak preparedness and response plans as a cross-cutting intervention in the context of low- and middle-income countries. We brought together civil society representatives, key stakeholders, and public health experts to explore areas of MHPSS integration during outbreak response.

**Results:**

Systematic integration of MHPSS into outbreak response was perceived to be feasible. Study participants strongly agreed that MHPSS can be integrated into most of the outbreak response pillars including partner coordination, case management, infection prevention and control, staff health and well-being, and risk communication and community engagement. However, the effort requires multi-sectoral collaboration, political commitment, and adequate recognition in planning and financing.

**Conclusions:**

Despite complex challenges, integrating MHPSS into outbreak pillars is possible. Moreover, emphasis should be placed on cultural adaptation of MHPSS guidelines and strong leadership in coordinating MHPSS into outbreak planning and response.

## Highlights

• Outbreaks have a considerable negative impact on mental health.
• Mental health issues are often overlooked during outbreak response.
• Mental health and psychosocial support (MHPSS) could address mental health and psychosocial needs during outbreaks.
• Public health experts call for the prioritisation of MHPSS during outbreak response.

## Introduction

Infectious disease outbreaks and other public health emergencies have significant health, economic, and societal impact. The socio-economic effects of outbreaks, such as food insecurity, social isolation, and forced displacement [1], cause emotional distress, anxiety, depression, and severe mental health conditions, with suicide documented as a particularly serious effect [2,3]. These effects are experienced in different ways across people and communities, causing social inequalities, especially among vulnerable groups such as people with pre-existing mental health conditions [4]. Additionally, measures taken to control the spread of outbreaks can contribute to negative effects on the mental health of the affected community. For instance, social distancing and quarantine measures lead to increased risk of adverse mental health effects such as depression, anxiety, and other stress-related conditions [5]. At the same time, the spread of outbreaks overwhelms health systems and disrupts the delivery of regular health services to people with existing mental health conditions [2,6].

Outbreaks are known to be associated with increased prevalence of mental health conditions. This was recorded during several epidemics, such as the COVID-19 [7,8], Ebola [9], and SARS epidemics [10]. A follow-up study found that a third of those diagnosed with COVID-19 developed a psychiatric or neurological condition within 6 months [11], and the incidence rate increased to almost 50% in those admitted to intensive care units. It was also reported that people with mental health conditions are more likely to contract COVID-19, to have severe symptoms, and to have higher mortality from COVID-19–related complications compared with the general population [12,13].

Despite the increasing risk of mental health conditions during outbreaks [13,14], the current public health response efforts in most countries provide insufficient mental health and psychosocial support (MHPSS) [3]. The role of MHPSS has been overlooked in outbreak preparedness and response plans, yet mental well-being is crucial to promoting resilience and rebuilding affected communities [15,16]. MHPSS interventions can take many forms but usually refer to “any type of local or outside support that aims to protect and promote psychosocial well-being and/or prevent or treat mental disorders,” according to the definition of the global coordinating mechanism for MHPSS, the Inter-Agency Standing Committee (IASC) [17].

It has been suggested that MHPSS components can be integrated into most outbreak response pillars such as partner coordination, case management, maintaining essential health services, infection prevention and control (IPC), staff health and well-being, and risk communication and community engagement (RCCE) [18]. Noting this, the IASC Reference Group for MHPSS in emergencies developed the MHPSS Minimum Service Package to be used as global guidance for planning, implementation, and evaluation of MHPSS in emergencies, including outbreaks. Although normative guidance is available, there are several challenges that hinder the planning and implementation of integrated MHPSS service during outbreaks, particularly in low- and middle-income countries (LMICs).

This study aimed to identify the areas of outbreak response into which MHPSS can be integrated and explore the challenges that hinder the delivery of integrated MHPSS services during outbreaks. The study is based on the experience and views of civil society, people with lived experience of mental health conditions, global public health experts, and other key stakeholders in this field.

## Methods

### Study design

We used a participatory qualitative study design with focus group discussions (FGD) and a consensus workshop. We ran a virtual FGD with experts with lived experience from different LMICs. We also conducted a consultation workshop with representatives of civil societies, public health experts, and key stakeholders in infectious disease outbreaks and MHPSS.

### Data collection

#### FGD with people with lived mental health experience

People with lived experience of mental health conditions were recruited through the Global Mental Health Peer Network (GMHPN) [19]. These individuals actively engaged in advocacy initiatives, and collaborated with the World Health Organization (WHO) on RCCE activities. We contacted GMHPN explaining the aims and objectives of the study, seeking their support in facilitating the FGD and the recruitment of people with lived mental health experiences. Inclusion criteria were being a member of the GMHPN, age >18 years, understanding English, and willingness to participate.

The FGD took place through an online virtual meeting in April 2023. It was facilitated by the research team, using an interview topic guide with probing questions focused on the integration of MHPSS in national responses to outbreaks, provision of targeted support for individuals experiencing distress or mental health conditions, and continuity of safe and accessible mental health services provision across different phases of the pandemic. The FGD was conducted in English and lasted for 2 hours. The FGD was recorded (after obtaining approval of all participants) and transcribed in English for coding and analysis.

#### Consultation with global infectious diseases and mental health experts

The consultation workshop was held in Cairo, Egypt in May 2023. The participants were invited and selected on the basis of having relevant roles and extensive experience in strategic planning and response for infectious disease outbreaks in LMICs. We invited a wide range of stakeholders such as United Nations agencies, regional and national public health agencies, non-governmental organisations (NGOs), service users’ organisations, and academia and government entities for this workshop. We included participants who held relevant positions in these agencies for at least 1 year, worked in outbreak preparedness and response, understood English, and agreed to participate.

On the 1^st^ day of the workshop, representatives of different agencies and service user representatives presented content on past experience of infectious disease outbreaks, emphasising aspects related to mental health and well-being. These experiences were derived from LMICs. The subsequent sessions were structured around outbreak response pillars, aiming to understand how MHPSS integration could take place within these pillars, to address integration challenges and formulate recommendations. Participants were divided into three mixed groups, taking into account their diverse background, experience, and geographical representation. Later, they were invited to share their experiences and lessons learned regarding the integration of MHPSS into outbreak response, the challenges that they encountered, and examples of good practices from different contexts. The group facilitator moderated the discussion, while groups used a virtual whiteboard platform to capture the evidence shared during the discussions. In each session, group representatives presented the key results of the group discussion to the other groups for additional feedback and insights. The study team were also taking notes during group and panel discussions.

At the end of the workshop, the researchers organised the notes and transferred them into a spreadsheet to be included in the final analysis.

### Data analysis

The data were exported into NVivo 12 software (Lumivero), coded, and analysed by combining inductive and deductive approaches. Initially, we conducted forward coding to identify patterns, themes, and concepts from the data. Then, we used deductive content analysis to check how the concepts fit into the established outbreak response frameworks. We used the outbreak response pillars taken from WHO's Emergency Response Framework [20] to present stakeholders’ perspectives regarding the integration of MHPSS into outbreak response, focusing on necessary actions to improve infectious disease outbreak response. This systematic approach helped us understand the interplay between response inputs and achieved outputs.

## Results

### Participant profile

A total of nine members of the GMHPN participated in the FGD, sharing their insights and perspectives regarding MHPSS services in various infectious disease outbreaks within their respective countries. They were from Bangladesh, Egypt, India, Kenya, Malawi, Nigeria, South Africa, Uganda, and Zambia. A total of 27 people participated in the consultation workshop in Cairo. The workshop participants represented key partner organizations including the Africa Centres for Disease Control and Prevention, WHO Department of Mental Health, Brain Health and Substance Use, WHO Regional Office for the Eastern Mediterranean, WHO Lebanon country office, UNICEF, Egyptian Red Crescent, and the Egyptian Ministry of Health and Population. Moreover, public health researchers, key decision makers, and national planners on outbreaks and MHPSS also participated in both events.

### Integrating MHPSS into outbreak response pillars: challenges and considerations

#### Challenges in integrating MHPSS into outbreak response pillars

**Figure 1:**
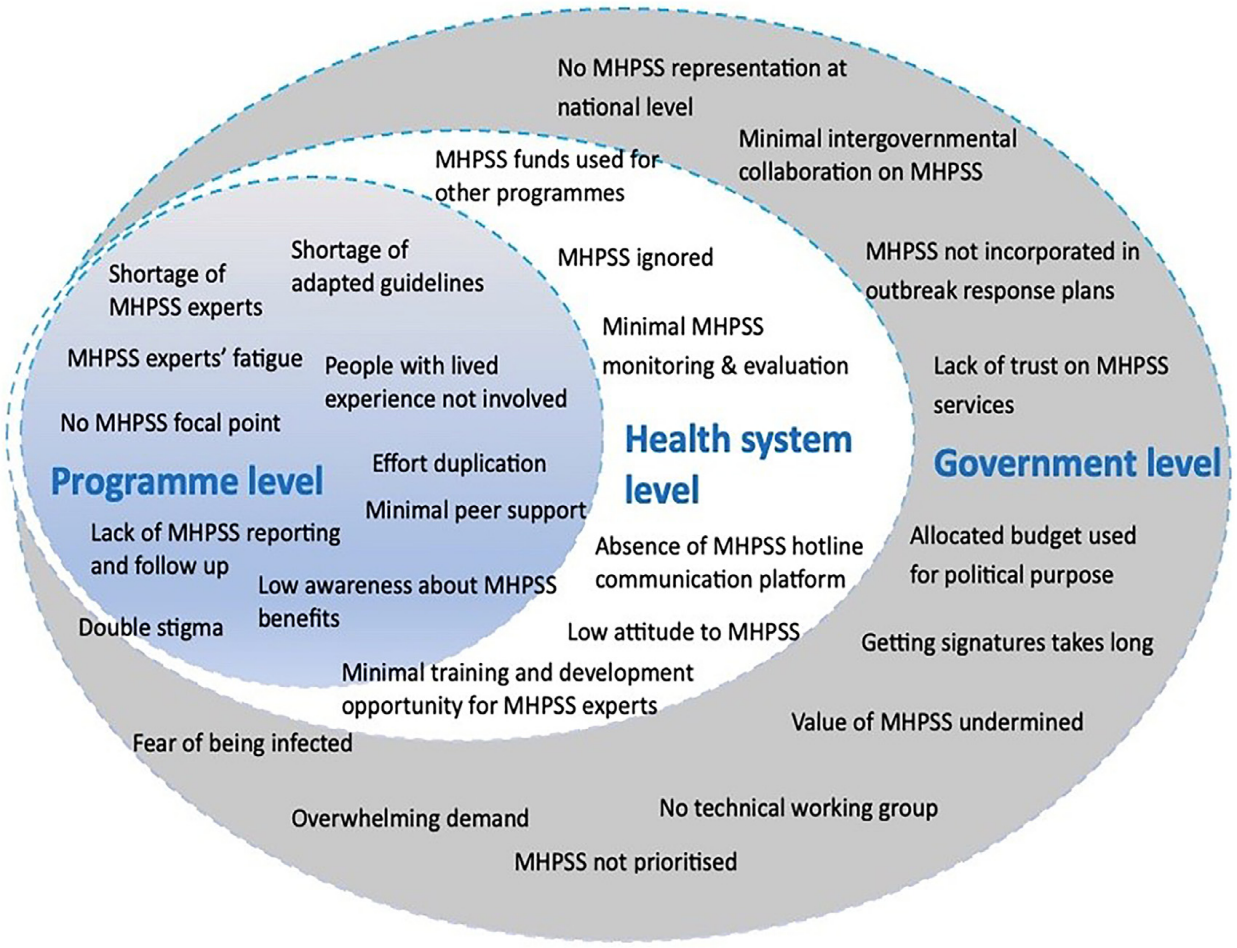
Conceptual model of barriers and challenges limiting integration of MHPSS into outbreak response. MHPSS, mental health and psychosocial support.

Study participants agreed on the importance of integrating MHPSS into the outbreak response and explored what considerations should be noted during outbreak preparedness and response efforts. They expressed thoughts about challenges based on the experience from previous outbreak responses (Fig. 1).

#### Coordination

Coordination was mentioned as a vital component of outbreak preparedness. It facilitates collaborations between different actors to avoid duplication, address obstacles, and fill gaps in the provision of services, and thus promotes the best use of available resources. Participants expressed their concern about the lack of coordination in outbreak response and how it could lead to a waste of resources.

“*There was no coordination of efforts between the ministry of health and NGOs. … there was poor coordination mechanism between the public health sector and the private agencies*.” (Participant from Eastern Africa)

In contexts where coordination mechanisms exist, people with lived experience are often not part of these coordination groups. Involving representatives of people with lived experience in this coordination would ensure that MHPSS activities are responsive to the needs of the target population. MHPSS coordination should be well-connected to the outbreak response coordination mechanism (e.g., Incident Management System) to ensure that MHPSS services are well-integrated.

#### Health service delivery

The de-prioritisation of mental health services was mentioned by participants from several countries. During infectious disease outbreaks, mental health services were significantly impacted, and often, other health services related to the outbreak were prioritised at the expense of mental health services.

“*With COVID-19, mental health stands on the sidelines, and facilities were rather used to look at the clinical side of infectious disease*.” (Participant from Southern Africa)

In some contexts, the importance of MHPSS was recognised after realising the significant impact of outbreaks on mental health.

"*It is only later in 2021, MHPSS started to be acknowledged. Because many people were not getting access to MHPSS, it was included lately*.” (Participant from Southern Africa)

The delivery and continuation of essential health services, including mental health services, is a vital component of outbreak response. Mental health services in this area include early identification and support to infected people who are experiencing psychological distress or mental health conditions, including services provided in communities, such as case management or social support services. In addition, the national clinical case management guidelines for the infectious disease should include MHPSS considerations. Health facilities need to be equipped with supplies of essential psychotropic medications. Crucially, essential psychotropic medication availability is often disrupted, and a continuous supply needs to be available, as the impact of abrupt discontinuation can be substantial for people with long-term mental and neurological conditions including epilepsy.

#### Infection prevention and control

Participants raised some issues related to IPC measures. For example, people in psychiatric hospitals and other residential facilities were disproportionately at risk of infection during outbreaks relative to others because of crowded spaces and difficult-to-implement IPC measures. This might also be exacerbated by the lack of access to IPC equipment such as masks and sanitisation in such facilities.

“*Those in psychiatric hospitals or other residential facilities were severely affected because they didn't have access to personal protective equipments*.” (Participant from Western Africa)

Another aspect of IPC that should be considered during outbreak response is the impact of IPC measures such as quarantine and isolation on mental health, so that these effects are well-understood and mitigation mechanisms can be established. For example, MHPSS experts need to be involved in awareness creation programmes to provide psychoeducation on the possible psychosocial effects of IPC measures, disseminate messages on coping strategies, and conduct targeted community sensitisation activities to reduce fears and change beliefs. In addition, MHPSS activities can be adapted according to IPC measures (e.g., by practicing physical distancing while promoting social interaction, including by using virtual platforms). When face-to-face intervention is necessary, such as in isolation treatment centres, hospitals, or quarantine facilities, MHPSS can be delivered in a way that promotes infection control (e.g., by using personal protective equipment or outdoor spaces). In some outbreaks where the body of the deceased person is infected, MHPSS could play a significant role in supporting the family of the deceased, allowing them to mourn and ensuring safe and dignified burial practices.

#### Risk communication and community engagement

Integrating MHPSS into RCCE is paramount to address the needs of the community. This should include disseminating key MHPSS-related information (e.g., on accessing available services or strategies for promoting well-being) and accurate and timely information about the outbreak through multiple media channels.

Stigma and misinformation are challenging issues during infectious disease outbreaks that increase fear and anxiety and could hinder access to health services for both mental health conditions and infectious diseases.

People who have an existing mental health condition experience double stigma when infected during an outbreak, and are sometimes excluded from proper health care because of the stigma of mental health conditions (called “diagnostic overshadowing”). Participants felt that stigma could worsen because of misinformation and inaccurate messaging about outbreaks.

“*Stigma was a double battle because you are stigmatised because of the mental health problem and now with COVID. This was especially because of misinformation provided about COVID. There was a lot of inaccurate information among the public*.” (Participant from Southern Africa)

Participants suggested that integrating MHPSS into RCCE can help address the double stigma associated with mental health conditions and infection through targeted messages, community dialogue, mobilisation, and sensitisation. They also stressed that RCCE activities should meaningfully engage affected communities, especially with people with lived experience, and other marginalised groups. Using social media and online platforms for RCCE activities was considered helpful, especially for youth groups, but there was a concern that certain population groups would not benefit from the same approach, such as older people and those who do not have access to the internet. Furthermore, there is a need to tailor messages for different groups and choose the appropriate platforms.

“*During COVID, we have seen a lot of initiatives on MHPSS about how to stay well during lockdown, but it was only accessible to those who use social media. A lot of older people did not have that access because they do not understand a lot about social media*.” (Participant from Southern Asia)

#### Staff health and well-being

Frontline work during outbreaks is demanding, with stress and fatigue being common challenges, as well as fear of infection. Addressing the MHPSS needs of staff is fundamental to building a resilient team that can handle the physical, mental, and emotional challenges of this work environment. Participants recommended that frontline staff be supported through training on emotional health, self-care, coping strategies, and safety, and be provided with opportunities for social support (e.g., peer support through remote messaging, voice or video conferencing tools).

Participants also suggested that everyone involved in the response should be trained on basic psychosocial support skills such as psychological first aid to be able to provide support to others when needed.

### Considerations to improve the MHPSS response to outbreaks

#### The role of people with lived experience in outbreak preparedness and response

People with lived experience of mental health conditions played a vital role in mitigating the psychological effects of outbreaks in their communities. They were able to use innovative solutions to reduce the impact of health services disruption and improve access to mental health services. During the COVID-19 outbreak (later pandemic), people with lived experience organised themselves to provide psychoeducation and support for their communities in addition to supporting their peers.

“*We developed flyers to tell people in a more of self-care type. What we also did was to host an online peer support group, and this has been extremely helpful*.” (Participant from Southern Africa)

Despite being motivated, in many contexts, people with lived experience were not formally involved in outbreak response or decision-making. Participants highlighted the importance of involving people with lived experience and community representatives in outbreak preparedness and response to ensure that response activities are appropriate and contextually relevant.

#### Going beyond COVID-19

Participants discussed that during several outbreaks in the past, such as those of Ebola, cholera, Lassa fever, and Nipah virus, they did not receive as much attention, especially from international agencies, as during COVID-19. In some contexts, other outbreaks happened in the midst of COVID-19, but individuals did not receive as much support as they did during COVID-19. The situation is even worse in the case of outbreaks affecting marginalised populations.

“*Nipah virus is very prominent in some areas in Asia, and people who actually suffer from this disease do not have enough information if there's any mental health support for them*.” (Participant from Southern Asia)

Participants recommended that MHPSS be included in preparedness and response plans for other outbreaks and epidemics, with more targeted support to marginalised and vulnerable populations.

#### Preparedness and recovery

Participants reached a strong consensus on the importance of preparedness to ensure a prompt and effective response to population needs during emergencies. Incorporating MHPSS in all-hazards planning is one way of achieving this. Participants suggested training staff and volunteers involved in the outbreak response to provide MHPSS to the affected population and support their peers, mapping existing MHPSS services, and strengthening coordination mechanisms among MHPSS actors.

## Discussion

Systematic integration of MHPSS into outbreak preparedness and response is central to global health security in general, and essential in application of global guidelines such as the MHPSS Minimum Service Package [21]. In this study, we included community representatives, public health experts, and global humanitarian actors, who felt that MHPSS can be integrated in culturally acceptable ways through stakeholder engagement and a participatory decision-making process, as demonstrated by some effective practices during the COVID-19 pandemic [22]. However, the available MHPSS materials are often written either in a limited number of languages or lack cultural appropriateness. Lack of adapted MHPSS guidelines has been a key factor reported in previous studies [23,24] and still continues to be an issue in the delivery of basic MHPSS services in outbreaks. The other key factor that was discussed in depth was the double stigma regarding MHPSS in outbreaks. Stigma acts as a barrier, affecting how frontline workers perceive mental health problems and respond to them [25,26]. Outbreaks often exacerbate limited access to essential psychotropic medications in many countries where infectious disease outbreaks are common [15]. The link between mental health problems and IPC measures is not well-understood, and programme planners and implementers are unaware of the importance of integrating MHPSS during public health emergencies including outbreaks [24,27], or do not have good guidance on how to do this.

Several factors influence the integration of MHPSS into outbreak response at the health system level. In most LMICs, health systems are under-resourced. When funds are made available during outbreaks, they are allocated for more immediate health concerns despite the clear evidence available about the increased burden of mental conditions [28]. While RCCE can serve as a tool to deliver key MHPSS messages to the community, the effort to integrate MHPSS in RCCE platforms in outbreaks has been minimal [29]. Furthermore, in most LMICs, health systems have no RCCE platforms for MHPSS [30], such as telephone support hotlines, messaging systems, and educational or awareness materials about mental well-being in schools and communities.

To fully integrate MHPSS, a health system must prioritise staff health and mental well-being. This was strongly advocated by most participants, who said that a lack of strategies supporting staff hinders effective outbreak response [31]. Since outbreak response is overwhelming, frontline health care workers experience fear, frustration, stress, burnout, and more severe mental conditions [32]. Working for long hours, being away from family, and exposure to distressing events are common experiences of frontline outbreak responders [33]. To create a well-prepared health system for outbreaks, strong staff support mechanisms should be in place at the health system level. In terms of human resource availability, in most LMICS, there are no tangible professional development opportunities for MHPSS workers, which makes it difficult to retain and quickly deploy MHPSS experts in emergencies.

Health system programme governance has a key role in resolving competing priorities. Strong governance processes can ensure the successful integration of MHPSS using a clear monitoring, evaluation, and learning mechanism during outbreak response plans, although in many instances this too is lacking.

Other factors at the national government level include political will and commitment to implement health system reform [24,27]. While public sectors and civil society are involved in outbreak preparedness and response, governments lead the overall coordination efforts. Thus, MHPSS representation at the national level is vital to make key decisions and ensure equity in MHPSS during outbreaks [34]. A technical working group for MHPSS can act as a liaison across sectors at the national level, and promote coordination with government during emergencies. In fact, emergencies (including outbreaks) can provide the incentive and momentum for rapid progress in policy and investment in MHPSS. This has been the case in a wide range of emergencies, as documented in the WHO's “Building Back Better” resource [35].

Overall, our study findings support the need to better apply the existing global guidelines on MHPSS, such as the IASC Guidelines on Mental Health and Psychosocial Support in Emergency Settings [17] and the associated guidelines for planning, implementation, and evaluation of MHPSS in emergencies [36,37]. More recently, the MHPSS Minimum Service Package has been considered as the core reference, and includes the best practices for outbreaks [21].

This study has some limitations to be noted. These include the following: i) study participants/experts were selected purposively on the basis of their prior experiences and their roles in relation to outbreaks and MHPSS services, which might not address the perspectives of wider affected communities and key decision makers; and ii) this study did not explore what community-level behaviour change activities are required during outbreaks, and is thus unable to provide insights on this aspect, although it was identified as an important area by participants.

## Conclusion

The experts asserted that the burden of mental health problems during infectious disease outbreaks can be addressed through a coordinated and integrated approach. Despite complex challenges, integrating MHPSS into outbreak pillars is possible through efficient use of technical resources that are culturally adapted, funding mobilisation, and ensuring that frontline responders have basic competencies and awareness in MHPSS. The participants of this study called for integration of MHPSS into outbreak preparedness and response plans, and strong leadership in coordination of MHPSS within outbreak response.

## Declarations of competing interest

The authors have no competing interests to declare.
